# Editorial: Immunological therapies in pediatric cancers: a Latin American perspective

**DOI:** 10.3389/fonc.2025.1744458

**Published:** 2025-12-10

**Authors:** Alberto Olaya-Vargas

**Affiliations:** 1 National Institute of Pediatrics (Mexico), Mexico City, Mexico; 2 ABC Medical Center, Mexico City, Mexico

**Keywords:** pediatric cancer, Latin America, pediatric immunotherapy, anti-GD2 antibodies, haploidenitcal stem cell transplantation

Immunotherapy has reshaped the global landscape of pediatric oncology, emerging as one of the most transformative therapeutic domains for children with relapsed, refractory, or high-risk malignancies. From bispecific antibodies to cell-based therapies and immune-modulating agents, these strategies—once confined to highly specialized centers in high-income countries—are now expanding into low- and middle-income regions. Latin America, a region marked by scientific talent but persistent structural inequalities, has entered a crucial phase in the adoption, evaluation, and local development of immunological therapies for childhood cancers.

Despite economic, logistical, and regulatory challenges, a growing network of pediatric oncology centers across Mexico, Brazil, Argentina, Chile, and Colombia is advancing innovative models for implementing antibody-based therapies, post-transplant immune modulation, and emerging cellular therapies. These pioneering efforts not only address the clinical needs of the region but also generate evidence that reflects unique epidemiologic patterns, resource constraints, and population-specific disease biology.

This Research Topic, *Immunological Therapies in Pediatric Cancers: A Latin American Perspective*, brings together a series of original contributions that illuminate the opportunities, challenges, and breakthroughs shaping the regional immunotherapy landscape. Each article offers a window into how Latin American institutions are reimagining care pathways for children with cancer through immunologic innovation.

## 1 Highlights of the Research Topic

### 1.1 Blinatumomab-induced remission followed by haploidentical transplantation in pediatric relapsed/refractory pre-B ALL: a multicenter study in Mexico

This multicenter Mexican study demonstrates that the integration of blinatumomab as a bridge-to-transplant strategy can yield high remission rates and successful transition to haploidentical hematopoietic stem-cell transplantation (HSCT). The findings highlight both the feasibility and effectiveness of bispecific T-cell engager therapy in resource-limited settings while underscoring the growing regional expertise in haploidentical transplant platforms (Olaya-Vargas et al). 

### 1.2 High-risk neuroblastoma in Mexico: from multimodal therapy to immunotherapy—experience with the first patient treated with naxitamab

The introduction of anti-GD2 immunotherapy in Mexico marks a milestone in the treatment of high-risk neuroblastoma. This case-based report contextualizes the first use of naxitamab in the country, illustrating how rigorous multimodal management combined with targeted immunotherapy can be successfully implemented in Latin American pediatric oncology units. It lays essential groundwork for broader national expansion of anti-GD2 treatment (Olaya-Vargas et al.).

### 1.3 Haploidentical, matched-related, and matched-unrelated donor HSCT for pediatric acute leukemias during the early years of haploidentical implementation in a developing country with a large donor registry

This contribution provides a comparative experience across donor types during the initial phase of haploidentical transplant adoption. The study reveals encouraging outcomes for haploidentical HSCT, supported by the presence of a robust national unrelated donor registry. These observations reinforce the critical role of donor availability, local infrastructure, and early expertise in accelerating the growth of cellular therapy in middle-income countries (Seber et al.).

### 1.4 Post-stem cell transplant maintenance for pediatric acute leukemias: insights from a Brazilian institution

Post-transplant maintenance therapy is a rapidly evolving field, particularly in acute leukemias with high relapse risk. This Brazilian report provides real-world insight into maintenance strategies such as targeted agents, immune-modulating therapies, and precision-guided approaches tailored to relapse biology. The work reflects a growing Latin American commitment to extending curative potential beyond HSCT by adopting post-transplant immunologic interventions (Breviglieri et al.).

### 1.5 Very early remission and increased apoptosis with pentoxifylline in pediatric acute lymphoblastic leukemia

This innovative investigation explores the anti-inflammatory and pro-apoptotic properties of pentoxifylline as an adjunctive immunomodulatory agent in ALL. The demonstration of early remission and enhanced leukemic apoptosis invites further exploration of cost-effective, accessible immunologic strategies that may complement standard therapy in low-resource environments (Salceda-Rivera et al.).

## 2 Regional progress and remaining challenges

Taken together, the contributions in this Research Topic underscore a dynamic era for pediatric immuno-oncology in Latin America. Across diverse institutions, immunotherapy is increasingly viewed not as an imported technology but as a domain ripe for regional innovation, clinical leadership, and context-specific solutions.

However, significant challenges remain:

- Equitable access to high-cost immunotherapies.
- Regulatory adaptability for advanced biologics and cell-based products.
- Sustainable financing models compatible with public health systems.
- Manufacturing capacity for CAR-T and other cell therapies.
- Specialized training to support rapidly evolving clinical technologies.

## 3 A vision for the future

The rise of immunotherapy in Latin America is more than a scientific advancement—it is a movement toward health equity and therapeutic justice. As local experts implement and generate evidence for cutting-edge therapies, they redefine the global narrative of what is possible in pediatric oncology outside high-income settings.

This Research Topic stands as a testament to the resilience, creativity, and scientific commitment that characterize pediatric oncology across the region. It invites researchers, clinicians, policymakers, and advocates to work collectively toward a future in which every child with cancer—regardless of geography—has access to life-saving immunological therapies.

